# Clinicopathological factors associated with HER2-positive gastric cancer: A meta-analysis: Erratum

**DOI:** 10.1097/MD.0000000000009530

**Published:** 2017-12-29

In the article, “Clinicopathological factors associated with HER2-positive gastric cancer: A meta-analysis”^[1]^, which appeared in Volume 96, Issue 44 of *Medicine*, the corresponding author's address should appear as Jian Zhang, Department of General Surgery, Xiangyang Central Hospital, Hubei University of Arts and Science, No. 136 Jingzhou Street, Xiangyang 441021, Hubei, China (e-mail: 361048757@qq.com).
